# Changes in Domestic Energy and Water Usage during the UK COVID-19 Lockdown Using High-Resolution Temporal Data

**DOI:** 10.3390/ijerph18136818

**Published:** 2021-06-25

**Authors:** Tamaryn Menneer, Zening Qi, Timothy Taylor, Cheryl Paterson, Gengyang Tu, Lewis R. Elliott, Karyn Morrissey, Markus Mueller

**Affiliations:** 1 European Centre for Environment and Human Health, University of Exeter Medical School, Knowledge Spa, Royal Cornwall Hospital, Truro, Cornwall TR1 3HD, UK; timothy.j.taylor@exeter.ac.uk (T.T.); C.B.Paterson@exeter.ac.uk (C.P.); g.tu@exeter.ac.uk (G.T.); L.R.Elliott@exeter.ac.uk (L.R.E.); k.morrissey@exeter.ac.uk (K.M.); 2 Environment and Sustainability Institute, Penryn Campus, University of Exeter, Penryn TR10 9FE, UK; M.Mueller@exeter.ac.uk; 3 College of Engineering, Mathematics and Physical Sciences, Harrison Building, Streatham Campus, University of Exeter, North Park Rd, Exeter EX4 4QF, UK; zq224@exeter.ac.uk; 4 College of Engineering, Mathematics and Physical Sciences, Penryn Campus, University of Exeter, Penryn TR10 9FE, UK

**Keywords:** sensors, electricity usage, gas usage, water usage, COVID-19

## Abstract

In response to the COVID-19 outbreak, the UK Government provided public health *advice* to stay at home from 16 March 2020, followed by *instruction* to stay at home (full lockdown) from 24 March 2020. We use data with high temporal resolution from utility sensors installed in 280 homes across social housing in Cornwall, UK, to test for changes in domestic electricity, gas and water usage in response to government guidance. Gas usage increased by 20% following advice to stay at home, the week before full lockdown, although no difference was seen during full lockdown itself. During full lockdown, morning electricity usage shifted to later in the day, decreasing at 6 a.m. and increasing at midday. These changes in energy were echoed in water usage, with a 17% increase and a one-hour delay in peak morning usage. Changes were consistent with people getting up later, spending more time at home and washing more during full lockdown. Evidence for these changes was also observed in later lockdowns, but not between lockdowns. Our findings suggest more compliance with an enforced stay-at-home message than with advice. We discuss implications for socioeconomically disadvantaged households given the indication of inability to achieve increased energy needs during the pandemic.

## 1. Introduction

The COVID-19 pandemic has been associated with 3.1 million deaths worldwide [1,2], as of 26 April 2021, and has caused significant disruption to individuals, societies and economies [3]. In an effort to reduce the spread of the virus, the UK Government provided public health guidance, from 3 March 2020 onwards, to stay at home and to increase the frequency and duration of handwashing [4,5,6]. People were formally *advised* by the UK Prime Minister to stay at home from the evening of 16 March 2020 (advice period) [4]. A week later, the first full lockdown came into force on the evening of 23 March 2020, when people were *instructed* to stay at home (instruction period). 

Reduction in virus transmission rates is reliant on individuals’ compliance with the government guidance. Other than a reduction in transmission rates, there are few sources of evidence of compliance. However, changes in domestic utility usage patterns can be used to test for evidence of behavioural changes in response to the government guidance to stay at home. 

Public compliance with guidance in a disaster scenario depends on perception of personal risk and trustworthy communications of consistent public information [7]. Specific to virus outbreaks, loss of income is a common concern for not wishing to comply with voluntary quarantine [8]. For COVID-19, compliance increases with fear of the virus [9] and with the stringency of government actions [10]. It therefore seems likely that public compliance will increase following the instruction to stay at home, given a growing number of COVID-19 cases implying higher personal risk, repetition of consistent public guidance, the announcement of the furlough scheme (20 March 2020) [11] and the closure of more activities, such as social venues and schools (20 March 2020) [6]. 

More time indoors provides more time for appliance and water use and an increased need for heating, leading to increased electricity, gas and water usage when the house is occupied than when unoccupied [12,13,14,15,16,17,18,19]. During the containment measures in response to COVID-19 across a range of countries, national electricity consumption rates decreased overall, particularly on weekdays, due to lack of commercial demand [20,21,22]. However, studies of large cities have shown that domestic electricity distribution increased in the COVID-19 lockdown in spring 2020 in Lagos, Nigeria [23], and in New York’s lockdown, usage increased in the middle of the day [24] and self-reported usage shifted to later in the day [25]. During the UK lockdown, self-reported domestic activities shifted to later in the day, and revealed an increase in housework and food-related activities [26,27]. 

Detection of changes in utility usage would have implications for utility supply networks and on household expenditure. In the short-to-medium term, leading health organisations predict that COVID-19 will remain an active threat [28]. Further lockdowns have already been implemented in the UK since the first in spring 2020, and could also be used in the future. After the pandemic has passed, increased home-working is predicted to continue [29,30,31,32], thereby reducing energy demand in office settings and increasing or shifting domestic demand. With regards to household expenditure, increased energy poverty is seen as an unintended consequence of COVID-19 that requires targeted financial support [33]. The impact is likely to be greater in socioeconomically disadvantaged areas, such as the area for our study, which can have lower energy consumption than other areas [34,35], and are already experiencing a greater impact of COVID-19 more generally in terms of mental health, education and digital inequality [36,37,38]. Evidence for meaningful differences in utility usage during lockdown is therefore important in understanding the wide range of impacts of the COVID-19 lockdown [39]. 

The purpose of this study is to test whether domestic utility usage data changed as a result of the COVID-19 UK outbreak in spring 2020, by comparing the data during that time with data from the same dates in 2019. In contrast to previous research, reported above, our study uses data measured directly from the home, and includes gas and water usage, from a cohort of participants that spend a high proportion of time at home normally, and live in a semi-rural, deprived area of England. Data from 280 sensored homes are used, providing high temporal resolution, allowing us to test for changes in usage patterns across the day, as well as overall differences. 

Primarily, we test for changes in spring 2020, in two time periods under different levels of Government guidance. The *advice period* is defined as 17 to 23 March 2020, when people were advised by the UK Government to stay at home, prior to the full lockdown in the UK. The *instruction period* is defined as the first month of full lockdown, from the 24 March to the 23 April 2020, when people were instructed to stay at home [4]. 

Our hypotheses are as follows: (1) As a result of the UK Government guidance, there should be an increase in the time spent indoors and the amount of handwashing in 2020 compared with 2019, which we expect to detect through increases in energy and water usage. We expect any changes to be stronger in the instruction period than the advice period. (2) We also expect energy and water usage to vary over different times of day; for example, lower usage at night than in the morning. Variation across the course of the day is expected regardless of behavioural responses to the COVID-19 guidance. In other words, we should see variation in 2019 as well as 2020. (3) We expect that changes as a result of COVID-19 guidance may differ at different times of day, for two reasons. Firstly the home would generally be occupied equally at certain times of the day (e.g., night-time, evening), so there may be no change due to stay-at-home guidance at those times. Secondly, there may be shifts in daily behaviour patterns, as people’s behaviours adjust to more time at home. 

In addition to the primary investigation of the first lockdown in spring 2020, we also repeat the analyses to test for changes throughout the course of the pandemic, including further lockdowns in November 2020 and January 2021, as well as periods when the restrictions were relaxed. 

## 2. Study Background and Data

### 2.1. The Smartline Project

The Smartline and Smartline Extension projects are a six-year interdisciplinary research programme that began in 2017, funded by the European Regional Development Fund, South West Academic Health Science Network, Cornwall Council and HM Government. The project involves collaboration between the University of Exeter, Coastline Housing (a social housing provider), Volunteer Cornwall (a charity that supports individuals and communities through voluntary action), Cornwall Council and the South West Academic Health Science Network. 

Smartline has recruited over 300 households in properties that are owned and managed by Coastline Housing in the Camborne and Redruth area of Cornwall, South West UK. The overarching aim of the project is to explore and trial opportunities for technology to support people to live healthier and happier lives in their homes and communities [40,41,42,43,44]. 

### 2.2. Data Collection

As part of the Smartline project, survey, sensor and housing data were collected from 280 homes, following informed written consent. The large dataset was a completely unique combination of cross-sectional and time-series data, including household characteristics, health measures, environmental readings and utility usages. 

#### 2.2.1. Survey Data

Face-to-face surveys were conducted in participants’ homes in September 2017 to November 2018. Questions covered a range of topics, such as digital technology in the home, health and wellbeing and community cohesion. 

#### 2.2.2. Sensor Data

Electricity meters were installed in 280 Smartline homes, gas meters in 52 homes and water meters in 22 homes. Readings were recorded at a maximum frequency of every 3 min for electricity, and every 7.5 min for gas and water, dependent on transmission success from the sensor to the host system. Homes for installation of gas and water meters were previously selected using cluster analysis on factors that can influence utility usage, in order to capture a representative sample of homes [45]. 

Sensors were also installed in 281 homes to measure temperature in the living room and main bedroom, with readings recorded at a maximum frequency of every 3 min. External air sensors were placed outside some homes, including measurements of temperature, with readings at a maximum frequency of every 30 min. 

All sensors were manufactured by Invisible Systems Limited [46] and installed by the Blue Flame company [47] from October 2017 onwards, and will be in place until August 2022. See Table S1 in the Supplementary Materials for sensor models and accuracy information. 

## 3. Materials and Methods

We may expect overall differences between utility usage between 2019 and 2020; however, given that the home would generally be occupied equally between the two years at certain times of the day (e.g., night-time, evening), we also examined the 24 h profiles. Identification of any changes across different times of day is only possible given the high temporal resolution in the Smartline sensor data. 

Data for all sensors were linearly interpolated to a sampling rate of 60 min, providing a value for each hour in each period. The means of hourly values across the days in the advice period and instruction period were calculated for each home to provide a 24 h usage profile for each outcome measure. (Times of day were adjusted for changes between Greenwich Mean Time and British Summer Time.)

We used mixed linear regression for our analyses. In the following sections, we describe the variables, followed by the analysis method details. 

### 3.1. Predictor and Outcome Variables 

Predictor variables were year (2019 and 2020), period (advice and instruction) and hour of the day (0–23). Outcome variables were the mean electricity (kWh), gas (m^3^) and water (m^3^) usages per hour in each home, with separate regressions conducted for each measure. 

### 3.2. Covariates

The survey, sensor and housing data allow us to include factors in our analysis that may affect utility usage in addition to increased time spent indoors. Table 1 summarises the factors identified by previous research and provides the Smartline survey, sensor or housing data used to create the covariate measures for inclusion in the analyses. For the homes used in the energy analyses, we also considered heating type [35], but it was not included as a covariate because all except six homes had gas-powered heating. The remaining six homes had air source heat pumps, and were included in the electricity analysis. All homes with gas monitoring had gas heating. 

The following covariates were included where possible: household size, presence of occupants under 18 years of age or in employment, time normally spent indoors, presence of a smart meter, number of rooms, property type, fuel poverty survey score, mean indoor temperature, and IMD rank. Mean indoor temperature was calculated from time-series data sets for homes with corresponding utilities data sets. The electrical appliances measure was also included for the electricity analysis. IMD rank was excluded from the water analysis, given a variance inflation factor of 38.8 in combination with the other factors, and the water smart meter was not included, given all ‘No’ responses for those homes with a Smartline water meter. For the gas analysis, given the small sample size, only the repeated measures (year, period and hour) were included due to low numbers for the different levels of the independent measures. Variance inflation factors for all included covariates were below 3.9.

### 3.3. Datasets

Homes were excluded from the analysis if the relevant survey responses for covariates were missing, the participant withdrew from the study before 24 April 2020, the sensor had been removed for practical reasons or the data indicated a recording error. Specifically, homes were excluded if either year mean (2019 and 2020) was zero (5%), the year mean had either doubled or halved from 2019 to 2020 (28%), or data were missing from a given hour across all days in the period (32%). For electricity, three homes were also excluded due to a mean hourly usage below 0.08 kWh. Only a subset of Smartline homes were monitored for gas and water usage, as described in the Study Background, Section 2.2.2. Complete datasets comprised 50 homes for electricity, 8 for gas and 14 for water, with a minimum of 33,644, 13,457 and 13,014 original recordings per home, respectively. 

### 3.4. Regression Method

The year, period and hour of the day provide repeated measures within each home, while the survey factors and mean indoor temperature are independent measures. We therefore used a linear mixed effects regression to test for changes in usage with year (from 2019 to 2020). We also included the following interactions between variables, to test whether any changes with year differed over the different periods (advice and instruction) or hours of the day: year × period, year × hour and year × period × hour. The unique property reference number (UPRN) for all homes was included as a random effect to represent the change within each home, and to capture the repeated measures. Models were implemented using R version 4.0.2 [66] and the lmer function from R’s lmerTest package library [67], which uses the lme4 package [68]. 

For each outcome variable and each time period combination (both periods together, advice period separately and instruction period separately), three models were fit. The null model comprised an intercept and the UPRN random factor. The main effects model comprised all factors but not the interaction terms. The interaction model included all factors and the year × hour interaction term, and the model using data for both periods also included the year × period, period × hour and year × period × hour interaction terms. 

The plots of residuals against fitted values showed evidence of heteroscedasticity for all utilities and regression models. These plots became more evenly distributed when electricity data were natural-log transformed and gas and water were square-root transformed (see Figure S1 in the Supplementary Material). The results of the analyses with transformed data are therefore reported. The patterns of results with non-transformed data are similar, with differences noted in Section 4. The quantile–quantile plots of the residuals revealed generally symmetric and sufficiently normal distributions. 

For all outcome variables and period combinations, the main effects model was a significantly better fit than the null model (all *χ*^2^ > 95, *p* < 0.001), and the interactions model was a significantly better fit than the main effects model (all *χ*^2^ > 79, *p* < 0.001). The results of the interaction models are therefore reported. 

For each regression, a reference hour was defined as the hour in which the absolute difference between 2019 and 2020 was the smallest. This hour of the day represents the minimum change that occurred during lockdown, and acts as a comparison to test whether changes during lockdown are larger at other hours of the day. 

Even in cases of no significant interactions with period, separate analyses were also conducted for the advice and instruction periods. There were two reasons for planned stratification of the analyses. Firstly, the reference hour differed between the two periods for all utilities (see Tables S3–S5 in the Supplementary Material). Secondly, there was evidence for changes in all utility usages between the advice and instruction periods (see Section 4). 

## 4. Results

**Figure 1:**
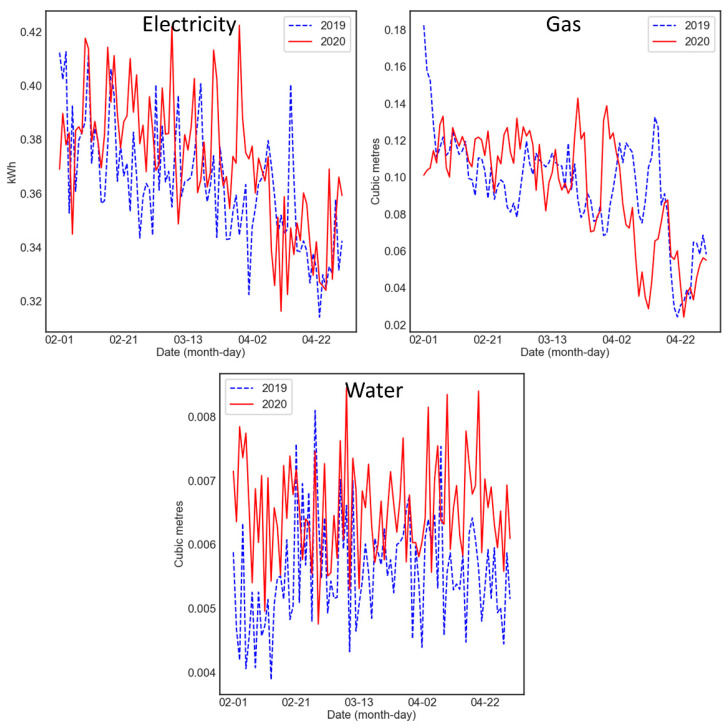
Mean hourly usage per day over all homes from February to April in 2019 and 2020 for electricity (**upper left**), gas (**upper right**) and water (**lower panel**).

Figure 1 provides an overview of the sensor data from February to April. Average hourly usage rates in the UK are 0.2–0.8 kWh for electricity, 0.08–0.18 m^3^ for gas and 0.008–0.016 m^3^ for water [69,70]. 

Table S2 in the Supplementary Material provides descriptive statistics for each covariate for each group of homes with electricity, gas and water usage measurements. Tables S3–S5 provide the detailed regression model outputs reported in the following subsections. 

The survey data, taken prior to the COVID-19 pandemic, show that the Smartline participants normally spend a high proportion of time at home. Of the Smartline participants, 59% responded that they were retired or long-term sick or disabled, while 17% were in full-time employment, and the mean time normally spent inside the home per day was 19.5 h. 

**Figure 2:**
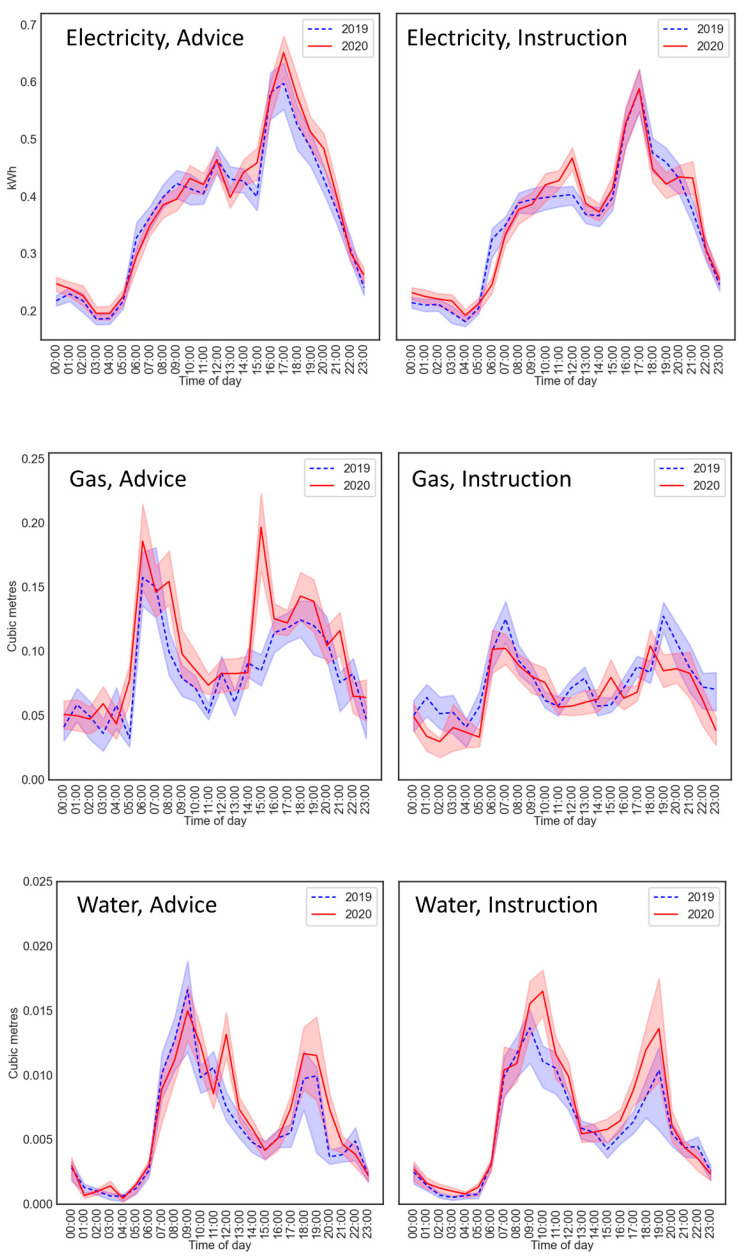
Electricity (**upper panels**), gas (**middle**) and water (**lower**) usage during the advice (**left**) and instruction (**right**) periods. Error bands represent 0.5 standard errors. The scale on the vertical axis applies to both panels of the same measure.

The 24 h profiles, averaged across homes, are shown in Figure 2. As anticipated, the pattern of the 24 h profiles indicates that there are differences across years, and that they may only be apparent at certain times of the day. These patterns indicate that rather than utility usage necessarily increasing among the Smartline cohort, the timing of everyday activities linked to utility usage shifted. Given the high temporal resolution of the Smartline sensor dataset, it was possible to identify any changes in usage across the time of day, by splitting the day into hourly sections for the analyses. 

### 4.1. Electricity

In the overall analysis, containing data from both periods, there was no significant relationship between electricity usage and year (*p* = 0.200), and no interaction with period (*p* = 0.169). The year × hour interaction was significant (*p* = 0.010), but the difference across years at each hour was not significantly different from the difference at the reference hour (all *p* > 0.117). Electricity usage decreased from the advice period to the instruction period (*p* = 0.006), and period interacted with hour (*p* = 0.008). The three-way interaction, year × period × hour, was not significant (*p* = 0.276). 

Electricity usage changed with hour of the day (*p* < 0.001), as would be expected. Hourly electricity usage increased by 23% with each extra person in the household (*p* = 0.030) and increased by 59% with a change from flat to house (*p* = 0.002). No other factors were significant predictors of electricity usage (all *p* > 0.182). 

With non-transformed data, the year × hour interaction only approached significance (*p* = 0.054), and compared with the reference hour, there was some evidence of an increase in electricity usage from 2019 to 2020 at 15:00, 17:00 and 20:00 (*p* = 0.058, 0.079 and 0.082, respectively). 

The results for the overall analysis provide little evidence for a change in electricity usage between 2019 and 2020, nor for differences between the advice and instruction period. However, given the interactions with hour and a different ideal reference hour for the two periods, we examined the two periods separately, which did reveal differences.

During the instruction period there was a significant interaction of year × hour (*p* = 0.001), which was not significant during the advice period (*p* = 0.375). Compared with the reference hour (17:00), following the government’s instruction to stay at home, there was an increase in electricity usage from 2019 to 2020 at 12:00 (*p* = 0.023), and a decrease at 06:00 (*p* = 0.005). All other relationships held as for the overall analysis (see Table S3 for details). 

**Figure 3:**
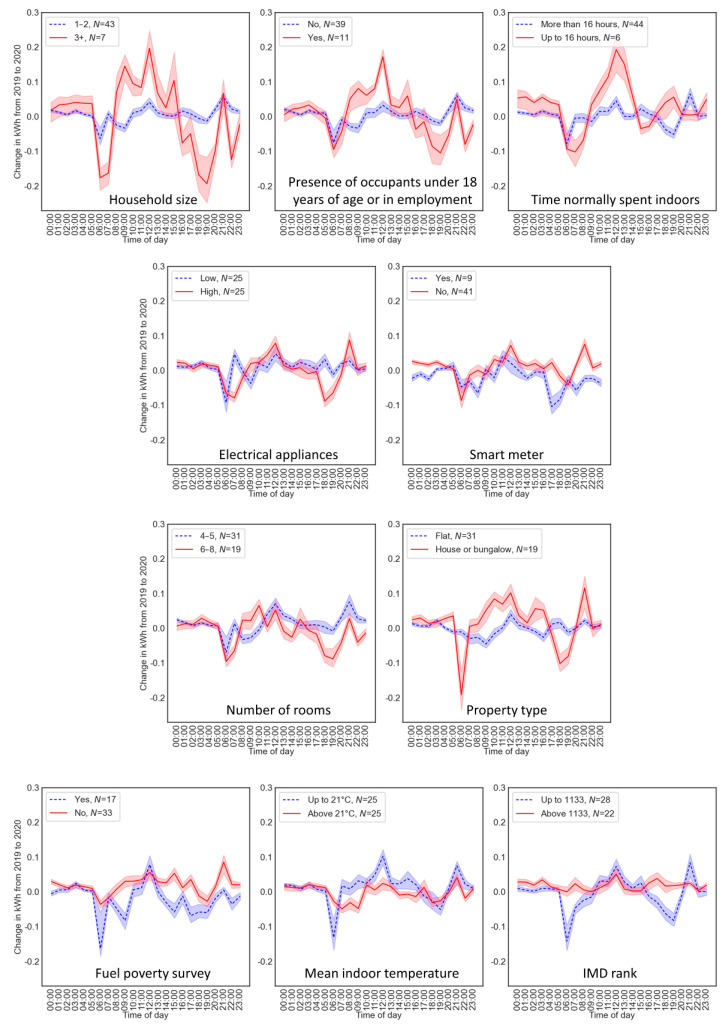
The change in electricity usage from 2019 to 2020 for households split into binary categories according to the values of the covariates. The group represented by the red line was considered more likely to be affected in terms of electricity usage by lockdown. Error bands represent 0.5 standard errors. See also Table S8 in the Supplementary Material.

We also examined the change in electricity usage from 2019 to 2020 using binary splits according to the values of the covariates. Figure 3 shows visual increases during the middle of the day for larger households, households with occupants under 18 years of age or in employment, households that normally spend less time indoors and houses or bungalows. Table S8 in the Supplementary Material shows the mean overall change from 2019 to 2020 for each covariate group. The largest increases between groups occurred for households without a smart meter, not suffering fuel poverty and with the highest IMD rank (least deprivation). Despite numerical and visual differences between the covariate groups, overall differences were not statistically significant (all *p* > 0.088; Table S8). 

### 4.2. Gas

In the overall analysis, there was no difference between years (*p* = 0.732), but there was a significant year × period interaction (*p* < 0.001), which is explored below, when the advice period and instruction periods were analysed separately. There was a trend towards decreased gas usage from the advice period to the instruction period (*p* = 0.068), and changes in gas usage with hour of the day (*p* < 0.001). All other interactions were not significant (all *p* > 0.173). With non-transformed data, the decrease with period was significant (*p* = 0.018) and the period × hour was significant (*p* = 0.014). 

During the advice period, hourly gas usage increased with year (*p* = 0.003) by 20% from 0.083 m^3^ in 2019 to 0.100 m^3^ in 2020, but there was no significant change in the instruction period (*p* = 0.136). Hour remained a significant predictor in both periods. 

### 4.3. Water

In the overall analysis, hourly water usage increased between years (*p* = 0.046) by 17% from 0.006 m^3^ in 2019 to 0.007 m^3^ in 2020, and increased from the advice period to the instruction period (*p* = 0.012). No interactions were significant (all *p* > 0.557). Water usage changed across hours of the day (*p* < 0.001). There was a trend towards a significant increase in water usage with each extra person in the household (*p* = 0.059). No other factors were significant predictors of water usage (all *p* > 0.228). With non-transformed data, period was not a significant predictor of water usage (*p* = 0.417) and the relationship with household size was significant (*p* = 0.013).

The results for the overall analysis show increased water usage in 2020 compared with 2019. Examination of the two periods separately revealed a trend towards year being a significant predictor of water usage in the advice and the instruction periods (*p* = 0.088 and 0.096, respectively). All other relationships were similar to the patterns in the overall analysis (see Table S5 for details). With non-transformed data, year was only a significant predictor of water usage during the instruction (*p* = 0.040), not the advice period (*p* = 0.162), and household size was a significant predictor of water usage in both periods (both *p* < 0.017). 

The regression coefficient estimates for the difference between years at each level of hour reflected the shifts in the morning peaks in both periods in Figure 2, with weak evidence for a greater increase at 12:00 in the advice period (*p* = 0.082), and a greater increase at 10:00 in the instruction period (*p* = 0.003). However, the year × hour was not significant for either period (both *p* > 0.543).

### 4.4. After the First Lockdown

The focus of this Special Issue is on the first responses to COVID-19 in March to April 2020. However, the data allow us to test for evidence of behaviour change in later lockdowns in England, and of sustained behaviour change between lockdowns. 

**Figure 4:**
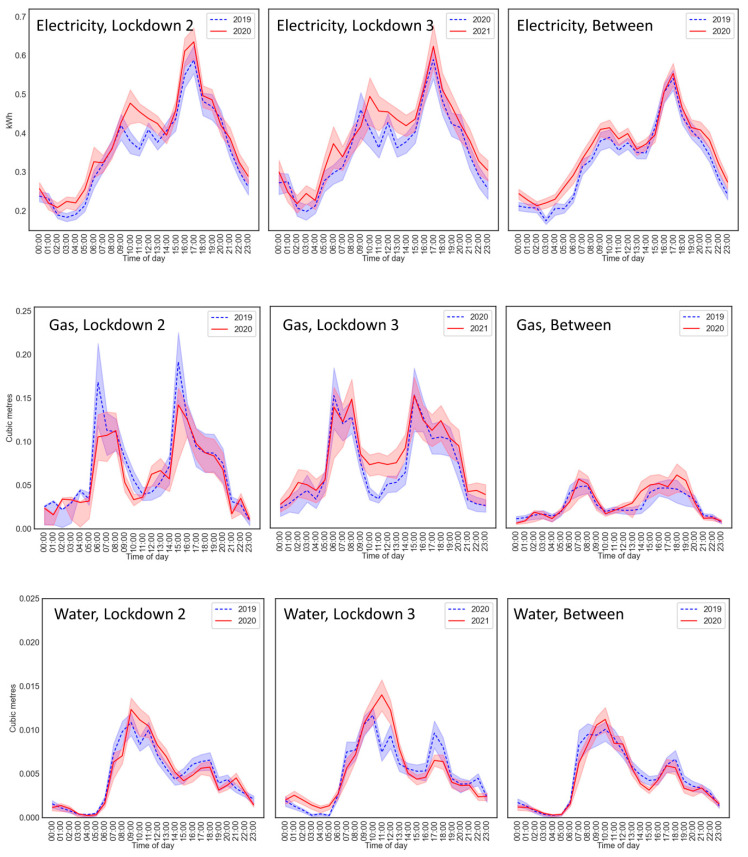
Electricity (**upper panels**), gas (**middle**) and water (**lower**) usage during the second lockdown (**left**), third lockdown (**middle**) and between lockdowns (**right**). Error bands represent 0.5 standard errors. The scale on the vertical axis applies to all panels of the same measure.

We repeated the analyses for the second lockdown from 5 November to 2 December 2020, the first month of the third lockdown from 5 January to 4 February 2021 and the time between lockdowns, from 1 September to 31 October 2020, during which many stay-at-home restrictions were lifted and most schools had reopened. Data from the 2021 lockdown were compared with data from the same dates in 2020. Data are presented in Figure 4. Outputs from the regressions are provided in Tables S6 and S7 in the Supplementary Material. 

Given the withdrawal of participants from the study and the removal of sensors in order to install smart meters, the sample sizes were smaller than for the first lockdown, with at least 21 for electricity, 6 for gas and 11 for water. We therefore do not statistically analyse the gas data. For homes with water meters, the presence of occupants under 18 years of age or in employment and the fuel poverty survey score were no longer included in the analyses due to raised variance inflation factors. 

#### 4.4.1. Second and Third Lockdowns

Unlike the first lockdown, electricity usage increased overall in both the second and third lockdowns compared with the previous year (both *p* < 0.039). There were no overall changes in water usage (both *F* < 1). 

As for the first lockdown, there was evidence that electricity and water morning usage shifted later in the day. For electricity, in the second lockdown, there was a stronger increase between years at 10:00 and 11:00 compared with the reference hour (08:00). However, the year × hour interaction was not significant (*F* < 1). For water, in the third lockdown, the interaction was significant (*F* = 1.857, *p* = 0.012), with a stronger increase at 11:00 (*p* = 0.023) and decrease at 22:00 (*p* = 0.041), compared with the reference hour (00:00). 

Visually, for gas, in Figure 4, there is some indication of reduced usage during the second lockdown compared with 2019 at some times of day, and for increased usage overall in the third lockdown. 

#### 4.4.2. Between Lockdowns

There was a trend towards increased electricity usage in 2020 compared with 2019 (*F* = 3.549, *p* = 0.067), and no significant difference for water (*F* < 1). The regression coefficients showed evidence for an increase in electricity from 02:00 to 05:00 (all *p* < 0.037, compared with the reference hour of 16:00). However, the year × hour interactions were not significant (*F* = 1.232, *p* = 0.207). For gas, Figure 4 shows slightly more gas usage in the afternoon in 2020 compared with 2019, although gas usage was lower at this time of year than in any of the lockdowns. 

## 5. Discussion

Utilising the Smartline Project network of 280 sensored homes, the purpose of this study was to test for changes in electricity, gas and water usages during the COVID-19 outbreak in the UK. We examined data from the week following advice to stay at home and from the month following the instruction to stay at home in 2020, and compared them with data from the same periods in 2019. Our hypotheses were: (1) increases in utility usages in 2020 compared with 2019, with any changes being smaller in the advice period than the instruction period; (2) differences in utility usage at different times of day, regardless of behavioural responses to the COVID-19 guidance; and (3) changes across years to interact with hour of the day. We found evidence to support all hypotheses, except that changes in gas usage were stronger during the advice than instruction period, perhaps due to changes in the weather condition. The changes in energy usage (gas and electricity) are discussed in the context of temperature, followed by the changes in water usage.

**Figure 5:**
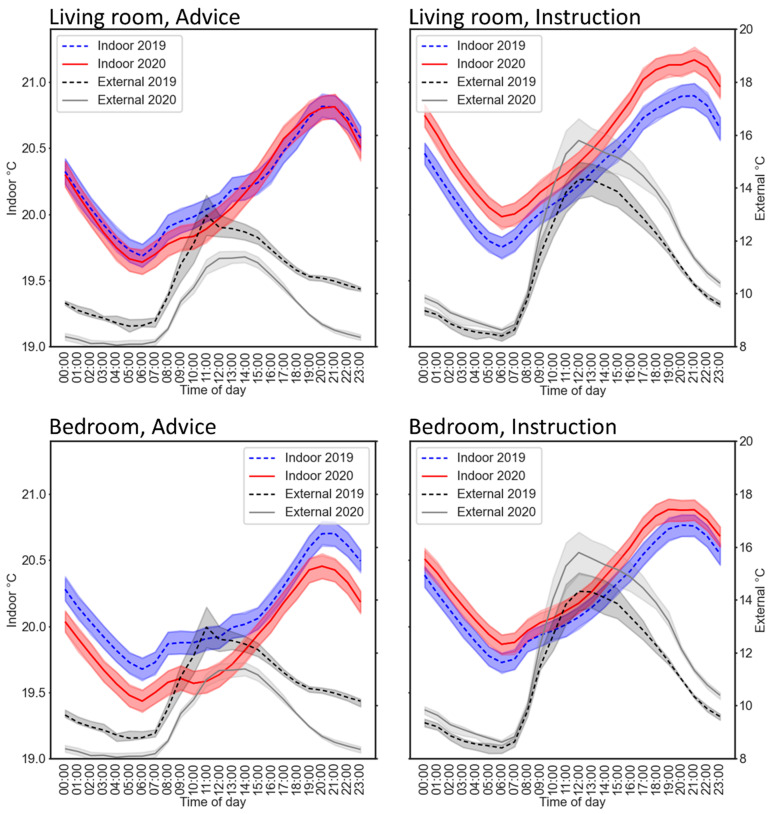
Red and blue lines show the indoor temperature in the living room (**upper panels**) and bedroom (**lower panels**) during the advice (**left**) and instruction (**right**) periods in 2019 and 2020. Grey lines show the external temperature during the same periods. Error bands represent 0.5 standard errors, and the number of homes included for indoor temperature was 162 to 170. The scales on the vertical axes apply to both left and right panels.

Gas usage increased during the advice period, suggesting people were acting on the government advice to stay at home. However, there was no change in gas usage during the instruction period. All homes with gas sensors have gas-powered heating, so the change between these two periods could reflect weather conditions at the time of year becoming warmer, when there was decreased need for heating. Heating requirements would account for the difference between 2019 and 2020 being apparent earlier in March (advice) but not later (instruction). External sensor data (see Figure 5) showed that the mean temperature during the advice period was lower in 2020 than in 2019 (9.3 and 10.6 °C, respectively), but was slightly higher during the instruction period (12.0 and 11.1 °C), which could also have contributed to increased gas usage in the advice period during 2020 when compared to 2019, but not during the instruction period in 2020 when compared to the equivalent time period in 2019. 

There was no evidence that electricity usage was affected in the advice period. However, during the lockdown instruction period, morning electricity shifted later in the day with no change in the evening. These shifts, together with the visible shifts in morning water usage to later in the day, suggest that more time at home leads to people getting up later in the day or being more leisurely, with delayed activities such as morning showers or breakfast. There is also a visual increase in usage during off-peak hours (see Figure 2: 1–4 a.m.). 

Differences between the advice and instruction period were also supported by the indoor temperature (Figure 5), with temperature being generally lower in 2020 than 2019 during the advice period, and higher during the instruction period in the living room, consistent with people spending more time at home during the instruction period. The rise in living room temperature from 2019 to 2020 seems unlikely to be due to external temperatures, given the similar mean daily temperatures in 2020 (12.0 °C) and in 2019 (11.1 °C), and a smaller difference in bedroom temperature between 2019 and 2020. In addition, the indoor difference between 2019 and 2020 was fairly constant throughout the day, while the external difference varied, with similar minima at night and a larger difference between maxima during the day. 

Patterns in water usage provide further evidence that people were spending more time at home. Overall water usage increased, consistent with people following the guidance to stay at home and increase handwashing. One reason for increased water usage could be more gardening [26], which would be reflective of people staying at home, particularly with an extended period of warm, settled weather in the UK in spring 2020. Precipitation data show more rain in 2019 than 2020 (approximately 71 and 13 mm, respectively) [71]. Other possible reasons for the increase include people undertaking more cooking, eating and drinking at home, as also observed in self-reports of activities [26], especially with alternative venues such as cafes being closed. Self-reported activities during lockdown suggest a decrease in washing and showering [26], but people may have been taking more time in the shower [12], or having time for a bath instead of a shower, and using the lavatory more. With particular response to COVID-19, people should also be undertaking more handwashing and cleaning. 

Overall, there were differences in utility usages during the advice and instruction periods in March to April 2020 compared with the same periods in 2019. These changes may be less apparent than they might be in a general population given that a large proportion of the Smartline cohort, as an older demographic, spend a lot of their time inside the home, regardless of COVID-19. The findings for electricity are in line with previous research from large cities in countries other than the UK [23,24,25], and with self-reports of activities in the context of national electricity consumption in the UK during the spring 2020 lockdown [26,27]. Our results show that delayed or increased electricity usage is also observed in the UK, in a semi-rural area with above-average levels of deprivation. 

The delayed morning electricity and water usage suggest that people exhibited more compliance given the instruction to stay at home than given the advice to stay at home in the week prior to the full lockdown. Such behavioural change supports findings from previous research that compliance increases with fear of the virus and with stringency of the government guidance [10]. 

Behavioural changes indicated by the changes in utility usage patterns also appeared in the second and third lockdowns in England, with continued evidence for morning usage of electricity and water shifting to later in the day, and increased overall electricity usage. The overall increase in these lockdowns, but not the first lockdown, could be due to these later lockdowns occurring in winter months, as opposed to the first lockdown occurring in spring 2020 with notably fine weather. The patterns observed in the lockdowns were not present in the data between lockdowns. These further analyses suggest people were complying with the stay-at-home message during the second and third lockdowns, but that this behaviour was not sustained between lockdowns despite the continued threat of COVID-19 and general government advice to limit outdoor activities. 

We provide evidence that there were meaningful increases and temporal shifts in energy and water usage in the home following the UK Government COVID-19 guidance to stay at home. 

These findings are important for the wider impacts of the COVID-19 lockdown, particularly given predictions that COVID-19 will remain an active threat in the population in the short-to-medium term [28]. Our future research will examine the time course of effects throughout the pandemic in relation to the COVID-19 case rates reported by the media and the UK Government. We wish to determine whether there was increased compliance with government guidance at times of high case rates. Such a finding would support work showing that perceived risk increases compliance with instructions during disasters [7]. 

In addition, this study shows that behaviour change can be identified in time-series sensor data, which allow examination of the temporal shifts. In future developments, changes and anomalies could be identified to detect behaviour change relating to health, for example, someone becoming unwell or having a fall, or fuel poverty, with restricted energy usage. 

This study also has implications for other areas of research. Increased costs associated with higher levels of domestic utility usage may be seen as an unintended consequence of COVID-19 lockdowns. Here, we did not assess the impacts on expenditures, but in other assessments bills were predicted to increase by 10 to 30% while working at home [26,72]. The Smartline cohort resides in social housing and in a region with higher than average rates of deprivation, with all but three of the participating homes being in the lowest 40% of the most deprived areas in England (Index of Multiple Deprivation (IMD) ranks) [64]. Our findings indicate that within our cohort of households, economic and wider socioeconomic factors were observed in utility usage, with less deprived households, as measured by fuel poverty, using more electricity during the first lockdown (Figure 3). These patterns suggest that the increase required by other households during lockdown was not achievable by these homes. The impact of increased utility bills, or of the ability to afford required energy, could be greater on those in socioeconomically disadvantaged areas. These homes are already suffering other inequitable impacts from COVID-19 and the UK lockdown, such as an increased impact on mental health due to lack of space at home [36], future consequences of reduced school contact for pupils in state-funded versus privately funded schools [37] and digital inequalities [38]. 

In the context of energy savings and potential solutions for mitigating increased energy usage, Figure 3 shows that, without an electric smart meter, there are visual increases from 2019 to 2020 during the instruction period at 12:00 and 21:00, as also seen in Figure 2. The presence of a smart meter, including shower water use displays, can encourage reductions in energy consumption and water consumption [51,52,53]. Spending more time at home would increase the accessibility of usage information, thereby facilitating responses to it. While the effects are varied [52], with maintained engagement with the smart meter depending on several factors, one of the strongest motivations appears to be financial benefits [54,55], which would be relevant to homes struggling with the financial effects of locked-down time at home. 

More broadly, these changes in utility usage have implications for the supply and pricing of energy and water, and for consideration of the environmental impact from domestic usage. Changes in the temporal demands for domestic energy could affect peak loads and time-of-use or dynamic pricing [73], or encourage off-peak pricing policies for low-income households. This research supports proposals for considering the future sustainability of home domestic energy sources [74], especially given that time spent at home is likely to remain above the levels before the COVID-19 pandemic. In the short-to-medium term, time at home will be impacted by future lockdowns, and record-level job losses [75]. In the longer term, increased time at home will continue, with home working predicted beyond COVID-19 restrictions [29,30,31,32]. Energy demand is therefore likely to decrease in offices and increase or shift in domestic settings [76]. 

## 6. Conclusions

During the time that people were advised (17 March 2020) and then instructed (24 March 2020) to stay at home during the COVID-19 lockdown in 2020 in the UK, gas, electricity and water usage patterns changed, compared with the same time periods in 2019. Gas usage increased following the advice to stay at home, prior to the full lockdown instruction, which may in part reflect a change in the external air temperature. Following the lockdown instruction to stay at home, electricity usage shifted from morning to midday, while evening usage remained the same. Water usage increased overall, and peak usage shifted to at least one hour later in the day. The changes are consistent with people getting up later, spending more time at home and washing more. These findings provide evidence for behaviour change in response to the UK Government’s instruction to stay at home during the COVID-19 outbreak, but only weak evidence for people following the advice to stay at home prior to the instructed lockdown. 

Electricity and water usage data also provide evidence for such behavioural change during the second and third lockdowns in England, but virtually no evidence of sustained change during the time between these lockdowns. 

We show meaningful increases in utility usage during the UK COVID-19 lockdown, even though our participants normally tend to spend a high proportion of time at home. Such increases in utility usage will have an economic impact on households, or could be unachievable for those already in fuel poverty. These impacts seem particularly likely to affect those in socioeconomically disadvantaged areas, which are already suffering other inequitable impacts from the virus and unintended consequences of the lockdown.

## Figures and Tables

**Table 1:** Factors that can affect domestic utility usage, and the covariate measure created from the survey, sensor or housing data.

Factor Affecting Utility Usage and Supporting Literature	Survey Question or Source of Data	Survey Response Options (All Questions Also Had the Option to Not Answer)	Measure(s) Created	Missing Data
The number of the people in the household [13,15,16,17,18,19,48,49].	Please tell us the number of people in your household.	Numbers of males and females in the following age ranges: 0–12, 13–17, 18–65, 65+ years.	The total number of people living in the home.	No missing responses.
The UK lockdown would have particularly affected people under the age of 18 and those in employment due to closures of schools and places of work, thereby increasing the time spent at home. In particular, children and adolescents in the home can affect utility usage [16,48].	Please tell us the number of people in your household.	Numbers of males and females in the following age ranges: 0–12, 13–17, 18–65, 65+ years.	Set to 1 if the response for 0–12 or for 13–17 is greater than zero.	No missing responses for number of children and adolescents. For employment, cases with missing responses were excluded from the analyses. The two factors were summed to give a value of 0, 1 or 2, reflecting the potential effect of the lockdown on the individuals in the household.
	Last week, were you: (Include any paid work, including casual or temporary work, even if only for one hour.)	Working as an employee? Self-employed or freelance? Working paid or unpaid for your own or your families business? Away from work ill, on maternity leave, on holiday or temporarily laid off? Doing any other kind of paid work? On a government sponsored training scheme? Waiting to start a job you have already obtained? Actively looking for work? Retired (whether receiving a pension or not)? A student? Looking after home or family? Long-term sick or disabled? None of the above?	Employment was 1 if ‘working as an employee’, and 0 for all other non-missing responses.	
Time normally spent inside the home (see Section 1).	On average, about how many hours per day do you spend indoors at home during an average weekend day (including sleeping)? Question repeated for week day, and for your partner.	0 to 24	Mean time spent indoors, across the main respondent and his/her partner, and across week day and weekend day, weighted to give the average time spent at home each day.	Cases with missing responses were excluded from the analyses.
Electrical appliances [18].	Which of these pieces of technology do you have in your home and are they connected to the internet? (Select all that apply.)	Internet connection, Television, TV decoder (e.g., Sky, Virgin Media), Mobile phone, Computer, Tablet, Wearable technology (e.g., Fitbit), Smart watch, Other technology.	A measure of the electrical devices in the home. Count of the number of technology devices in the home, including those connected to the internet.	The survey question comprised a list with options to select, so missing responses were treated as a ‘No’ response.
Smart meters [50,51,52,53,54,55,56].	Does your home have smart meters for your energy/water supply?	No, Electricity, Gas, Water.	Whether or not the home has a smart meter for the relevant utility.	No missing responses.
The number of rooms in the home or floor area [13,16,17,18,19,35].	Please tick all the rooms that you have in your home.	Kitchen, Dining room, Utility room, Bathroom, Living room, Bedrooms 1 to 4, Other room.	Count of the number of rooms in the home.	No missing responses, except for ‘Other’, which was counted if it contained any text.
The building type [15,17,18].	Flat or house (including bungalow) obtained from Coastline Housing records.		Property type (flat or house).	No missing information.
Fuel poverty [48,57,58,59,60,61].	Do you think your home is adequately heated?	Yes/No	Combined to provide an indicator of fuel poverty. A score of 1 was assigned to ‘No’, ‘Yes’ and ‘Yes’, respectively, and summed to provide a score of 0 to 3. The fuel poverty measure was based on the definition “the state of being unable to afford to heat one’s home adequately” [62] (page: definition of fuel poverty), and on research showing that families suffering fuel and water poverty will change their behaviours, for example restricting heating and ventilation, to save energy and water [48,57,58,59,60,61].	Cases were excluded from the analyses if any response was missing.
	Do you avoid turning on the heating because of cost?	Yes/No		
	Do you avoid ventilating your home to save heat/energy?	Yes/No		
In addition to the fuel poverty measure constructed from the survey data, mean indoor temperature [63] and IMD rank [64] were also included as an indicators of fuel poverty [65].	Temperature data from Smartline living room and bedroom sensors.		The mean temperature over both rooms. Calculated from the mean of hourly values across the lockdown time period in both years to provide one value per home.	If sensor data was not present for both years, the case was excluded from the analyses.
	IMD rank using the postcode for the home.		606 to 19,024, with a lower rank indicating higher deprivation.	

## Data Availability

The majority of Smartline data are available by registering interest at www.smartline.org.uk/main-content-area/data-access (accessed on 8 January 2021).

## Supplementary Materials

The following are available online at https://www.mdpi.com/article/10.3390/ijerph18136818/s1, Table S1: Sensor information for each type of utility usage and air measurement, Table S2: Descriptive statistics for each relevant covariate, for each group of homes with electricity, gas and water usage measurements in the first lockdown, Table S3: Electricity usage regression model outputs for the first lockdown, Table S4: Gas usage regression model outputs for the first lockdown, Table S5: Water usage regression model outputs for the first lockdown, Table S6: Electricity usage regression model outputs for periods after the first lockdown, Table S7: Water usage regression model outputs for periods after the first lockdown, Table S8: Mean change in electricity usage from 2019 to 2020 in the instruction period of the first lockdown, split by binary categories according to the values of the covariates, Figure S1: Residuals as a function of the values fitted by the interaction regression model containing both periods, for non-transformed and transformed electricity, gas and water usage.
