# Supplementary material for: Changes in Domestic Energy and Water Usage during the UK COVID-19 Lockdown Using High-Resolution Temporal Data
Source: Int J Environ Res Public Health. 2021 Jun 25;18(13):6818. doi: 10.3390/ijerph18136818 (PMC8297134; doi:10.3390/ijerph18136818)
Supplement: Supplementary file 1 [file ijerph-18-06818-s001.zip › ijerph-1241165-supplementary.pdf]

Supplementary material:

Changes in domestic energy and water usage during the UK COVID-19 lockdown using high-resolution temporal data

Table S1: Sensor information for each type of utility usage and air measurement.

Sensor data type	Sensor model	Sampling interval	Measurement accuracy or operation notes
Electricity	ISL 067 Smart RF Mk2 3-phase wireless meter (ref: QC0142)	Readings recorded every 3 minutes.	±10%
Gas	Pulse transmitter (ref: QC0145b)	Count of the number of pulses generated per 7.5 minutes.	In line with equipment it is connected to.
Water	Pulse transmitter (ref: QC0145c)	Count of the number of pulses generated per 7.5 minutes.	In line with equipment it is connected to.
Indoor temperature	Ultra-RF forTemperature and humidity (ref: QC0160)	Readings recorded every 3 minutes. Update rate every 7.5 minutes.	±0.5°C
Outdoor temperature	Invisible Systems External AQ Monitor	Readings recorded every 30 minutes.	±0.3°C
Gateway	Boxed ISL058 gateway		RF transmitters connect to sensors and send data to the infrastructure internet connected Gateway on site. The Gateway stores and transfers the data to the Realtime Online cloud server over a secure mobile cellular connection.

Table S2: Descriptive statistics for each relevant covariate, for each group of homes with electricity, gas and water usage measurements in the first lockdown.

Notes: N is the number of homes in the group for the given measure.

	Electricity usage (*N* = 50)		Gas (*N* = 8)		Water (*N* = 14)	
Covariate	Mean (SD)	Min, max	Mean (SD)	Min, max	Mean (SD)	Min, max
Household size	1.7 (0.9)	1, 6	1.3 (0.5)	1, 2	1.5 (0.7)	1, 3
Under-18s or Employed	0.2 (0.5)	0, 2	0.1 (0.4)	0, 1	0.2 (0.4)	0, 1
Time indoors	20.5 (2.7)	12, 24	20.6 (3.3)	14, 24	19.9 (2.9)	13, 24
Electrical appliances	7.9 (3.1)	1, 13				
Electricity smart meter	0.2 (0.4)	0, 1				
Number of rooms	5.4 (1.0)	4, 8	5.6 (1.3)	4, 8	6.1 (1.2)	4, 8
Property type (0 = flat)	0.4 (0.5)	0, 1	0.1 (0.4)	0, 1	0.4 (0.5)	0, 1
Fuel poverty	0.5 (0.7)	0, 3	0.3 (0.5)	0, 1	0.4 (0.9)	0, 3
Indoor temperature	20.9 (2.1)	16.3, 25.5	22.2 (1.2)	20.8, 23.7	20.8 (2.2)	16.6, 23.7
IMD rank	2882.8 (3695.6)	606 ,11981	3297.4 (3668.6)	964 ,9855	3665.6 (4012.5)	606 ,10826

Notes for the following Tables S3 to S7*:*

Significance $^{+}p \leq 0.10$, $*p \leq 0.05$, $**p \leq 0.01$, $***p \leq 0.001$.

N is the number of homes in the group for the given measure; *NObs* is the total number of sensor data points used as the outcome in the regression that includes period as a factor = *N* x 2 years x 2 periods x 24 hours.

CE is the Coefficient estimate; *t* or *F* is the *t* value from the regression or, where noted, is an *F* value; *p* is the *p* value.

The CE provides the rate of change over different values of the variable. The reference year was 2019, so the CE represents the change from 2019 to 2020, and the reference period was advice, so the CE represents the change from the advice to the instruction period. The reference hour differed across regression models, and was chosen to be the hour at which there was the minimum absolute difference between the two years, which allows any differences between the years for each hour to be detected by the interaction.

Year, Period and Hour are repeated measures factors. *F* and *p* values are reported from the overall ANOVA for the interaction model. For Year and Period, coefficient estimates are reported from the main effects model given that the interaction model provides the estimate for the reference level of Hour. For Hour, the main effects regression model provides estimates for 23 levels versus the reference level, so estimates are not included. For the independent measures, all values (coefficient estimate, *t* and *p*) are reported from the interaction model.

In the main text, percentage increases in electricity usage with every unit increase in the predictor are reported for log-transformed outcomes, using CE to provide the multiplier, e^{CE}.

Table S3: Electricity usage regression model outputs for the first lockdown. N = 50; NObs = 4800.

	Both periods Reference Hour = 22:00			Advice period Reference Hour = 12:00			Instruction period Reference Hour = 17:00		
Predictor	CE	*t* or *F*	*p*	CE	*t* or *F*	*p*	CE	*t* or *F*	*p*
Year (reference = 2019)	0.031	$F = 1.687$	0.200	0.041	$F = 2.226$	0.142	0.022	$F = 0.852$	0.361
Period (reference = advice)	-0.028	$F = 8.429$	**0.006						
Hour		$F = 45.874$	***<0.001		$F = 41.156$	***<0.001		$F = 42.552$	***<0.001
Household size	0.210	2.259	*0.030	0.216	2.298	*0.027	0.203	2.202	*0.034
Under-18s or Employed	0.049	0.298	0.767	0.036	0.217	0.829	0.062	0.378	0.707
Time indoors	0.020	0.734	0.467	0.024	0.843	0.404	0.017	0.618	0.540
Electrical appliances	0.032	1.356	0.183	0.032	1.334	0.190	0.032	1.369	0.179
Electricity smart meter	0.081	0.468	0.642	0.101	0.576	0.568	0.061	0.356	0.724
Number of rooms	0.017	0.247	0.806	0.018	0.269	0.789	0.015	0.224	0.824
Property type (reference = flat)	0.463	3.280	**0.002	0.463	3.244	**0.002	0.462	3.294	**0.002
Fuel poverty	0.097	1.143	0.260	0.096	1.107	0.275	0.099	1.172	0.248
Indoor temperature	-0.010	0.308	0.760	-0.012	0.358	0.723	-0.009	0.256	0.799
IMD rank	0.000	0.579	0.566	0.000	0.668	0.508	0.000	0.483	0.632

Table S3 (continued): Electricity usage regression model outputs for the first lockdown.

	Both periods			Advice period			Instruction period		
Predictor	CE	*t* or *F*	*p*	CE	*t* or *F*	*p*	CE	*t* or *F*	*p*
Year x Period		$F = 1.894$	0.169						
Year x Hour		$F = 1.818$	**0.010		$F = 1.068$	0.375		$F = 2.165$	***0.001
Period x Hour		$F = 1.853$	**0.008						
Year x Period x Hour		$F = 1.155$	0.276						

Table S4: Gas usage regression model outputs for the first lockdown. N = 8; NObs = 768.

	Both periods Reference Hour = 14:00			Advice period Reference Hour = 12:00			Instruction period Reference Hour = 09:00		
Predictor	CE	*t* or *F*	*p*	CE	*t* or *F*	*p*	CE	*t* or *F*	*p*
Year (reference = 2019)	0.003	*F* = 0.128	0.732	0.027	*F* = 9.084	**0.003	-0.021	*F* = 2.830	0.136
Period (reference = advice)	-0.024	*F* = 4.658	+0.068						
Hour		*F* = 2.783	***<0.001		*F* = 2.917	***<0.001		*F* = 2.384	***0.001
Year x Period		*F* = 22.112	***<0.001						
Year x Hour		*F* = 0.921	0.570		*F* = 0.715	0.826		*F* = 0.961	0.518
Period x Hour		*F* = 1.279	0.174						
Year x Period x Hour		*F* = 0.810	0.720						

Table S5: Water usage regression model outputs for the first lockdown. N = 14; NObs = 1344.

		Both periods Reference Hour = 04:00			Advice period Reference Hour = 23:00			Instruction period Reference Hour = 21:00	
Predictor	CE	*t* or *F*	*p*	CE	*t* or *F*	*p*	CE	*t* or *F*	*p*
Year (reference = 2019)	0.003	*F* = 4.852	0.046	0.003	*F* = 2.933	$^{+}$0.088	0.003	*F* = 3.217	$^{+}$0.096
Period (reference = advice)	0.004	*F* = 8.603	0.012						
Hour		*F* = 18.751	***<0.001		*F* = 14.685	***<0.001		*F* = 19.620	***<0.001
Household size	0.034	2.331	$^{+}$0.059	0.036	2.450	*0.050	0.033	2.207	$^{+}$0.069
Under-18s or Employed	0.033	1.341	0.229	0.031	1.266	0.253	0.035	1.411	0.208
Time indoors	0.002	0.652	0.539	0.003	0.819	0.444	0.002	0.486	0.644
Number of rooms	0.001	0.111	0.915	0.000	0.039	0.970	0.001	0.182	0.861
Property type (reference = flat)	-0.001	0.027	0.979	0.002	0.072	0.945	-0.003	0.125	0.905
Fuel poverty	-0.008	0.584	0.580	-0.008	0.556	0.598	-0.009	0.610	0.564
Indoor temperature	0.004	0.631	0.552	0.005	0.717	0.500	0.004	0.543	0.606
Year x Period		*F* = 0.020	0.888						
Year x Hour		*F* = 0.930	0.558		*F* = 0.669	0.875		*F* = 0.941	0.544
Period x Hour		*F* = 0.569	0.949						
Year x Period x Hour		*F* = 0.738	0.809						

Table S6: Electricity usage regression model outputs for periods after the first lockdown.

	Second lockdown (N = 33) Reference Year = 2019 Reference Hour = 08:00			Third lockdown (N = 21) Reference Year = 2020 Reference Hour = 20:00			Between lockdowns (N = 43) Reference Year = 2019 Reference Hour = 16:00		
Predictor	CE	t or F	P	CE	t or F	p	CE	t or F	p
Year	0.057	F = 4.695	*0.038	0.086	F = 5.176	*0.034	0.074	F = 3.549	0.067
Hour (reference = 22:00)		F = 33.095	***<0.001		F = 16.741	***<0.001		F = 39.134	***<0.001
Household size	0.128	0.878	0.389	0.280	1.074	0.308	0.260	2.242	0.308
Under-18s or Employed	0.057	0.269	0.790	0.265	0.920	0.379	0.089	0.500	0.379
Time indoors	-0.005	0.120	0.906	-0.024	0.367	0.721	0.024	0.676	0.721
Electrical appliances	0.048	1.329	0.197	0.044	1.107	0.294	0.044	1.577	0.294
Electricity smart meter	0.002	0.007	0.994	0.211	0.737	0.478	0.046	0.270	0.478
Number of rooms	0.091	0.797	0.434	0.054	0.424	0.681	-0.069	0.738	0.681
Property type (reference = flat)	0.316	1.376	0.183	0.221	0.864	0.408	0.173	1.090	0.408
Fuel poverty	0.139	1.509	0.145	0.391	2.400	0.037	0.170	2.059	0.037
Indoor temperature	0.013	0.260	0.797	0.008	0.156	0.879	0.003	0.084	0.879
IMD rank	0.000	1.289	0.211	0.000	2.027	0.070	0.000	0.270	0.070
Year x Hour		F = 0.814	0.716		F = 0.773	0.766		F = 1.232	0.207

Table S7: Water usage regression model outputs for periods after the first lockdown.

		Second lockdown ($N = 12$) Reference Year = 2019 Reference Hour = 03:00			Third lockdown ($N = 11$) Reference Year = 2020 Reference Hour = 00:00			Between lockdowns ($N = 12$) Reference Year = 2019 Reference Hour = 05:00	
Predictor	CE	*t* or *F*	*p*	CE	*t* or *F*	*p*	CE	*t* or *F*	*p*
Year	-0.001	$F = 0.048$	0.830	0.002	$F = 0.667$	0.433	-0.001	$F = 0.664$	0.433
Hour		$F = 17.500$	***<0.001		$F = 13.276$	***<0.001		$F = 17.928$	***<0.001
Household size	0.006	0.398	0.704	0.000	0.029	0.978	0.002	0.141	0.892
Time indoors	-0.002	0.807	0.451	-0.002	0.856	0.431	-0.003	1.001	0.355
Number of rooms	-0.001	0.084	0.936	-0.002	0.256	0.808	-0.001	0.231	0.825
Property type (reference = flat)	-0.002	0.120	0.909	-0.002	0.096	0.927	-0.011	0.607	0.566
Indoor temperature	0.006	1.341	0.229	0.007	1.399	0.221	0.005	1.301	0.241
Year x Hour		$F = 0.798$	0.733		$F = 1.857$	*0.012		$F = 0.997$	0.469

Table S8: Mean change in electricity usage from 2019 to 2020 in the instruction period of the first lockdown, split by binary categories according to the values of the covariates.

Covariate	Criterion for binary categorisation into Groups A and B, with B considered more likely for electricity usage to be affected by lockdown.	Group A mean (*N*)<br>Figure 3 blue dashed lines	Group B mean (*N*)<br>Figure 3 red solid lines	Mann-Whitney *U* (*p*)
Household size	A: 1 or 2<br>B: 3 or more	0.006<br>(43)	0.000<br>(7)	138<br>(0.743)
Presence of occupants under 18 years of age or in employment	A: None known<br>B: At least one	0.005<br>(39)	0.007<br>(11)	217<br>(0.953)
Time normally spent indoors	A: More than 16 hours<br>B: Up to 16 hours per day	0.002<br>(44)	0.031<br>(6)	107<br>(0.474)
Electrical appliances	A: Up to 8<br>B: More than 8	0.009<br>(25)	0.002<br>(25)	278<br>(0.503)
Presence of a smart meter	A: Yes<br>B: No	-0.022<br>(9)	0.011<br>(41)	124<br>(0.131)
Number of rooms	A: 4 or 5<br>B: 6 to 8	0.013<br>(31)	-0.007<br>(19)	245<br>(0.322)
Property type	A: Flat<br>B: House or bungalow	-0.001<br>(31)	0.016<br>(19)	284<br>(0.834)
Fuel poverty survey score	A: 1 to 3<br>B: 0	-0.022<br>(17)	0.019<br>(33)	202<br>(0.108)
Mean indoor temperature	A: up to 21 °C<br>B: above 21 °C	0.013<br>(25)	-0.002<br>(25)	291<br>(0.677)
IMD rank	A: up to 1133<br>B: above 1133	-0.004<br>(28)	0.018<br>(22)	395<br>(0.089)

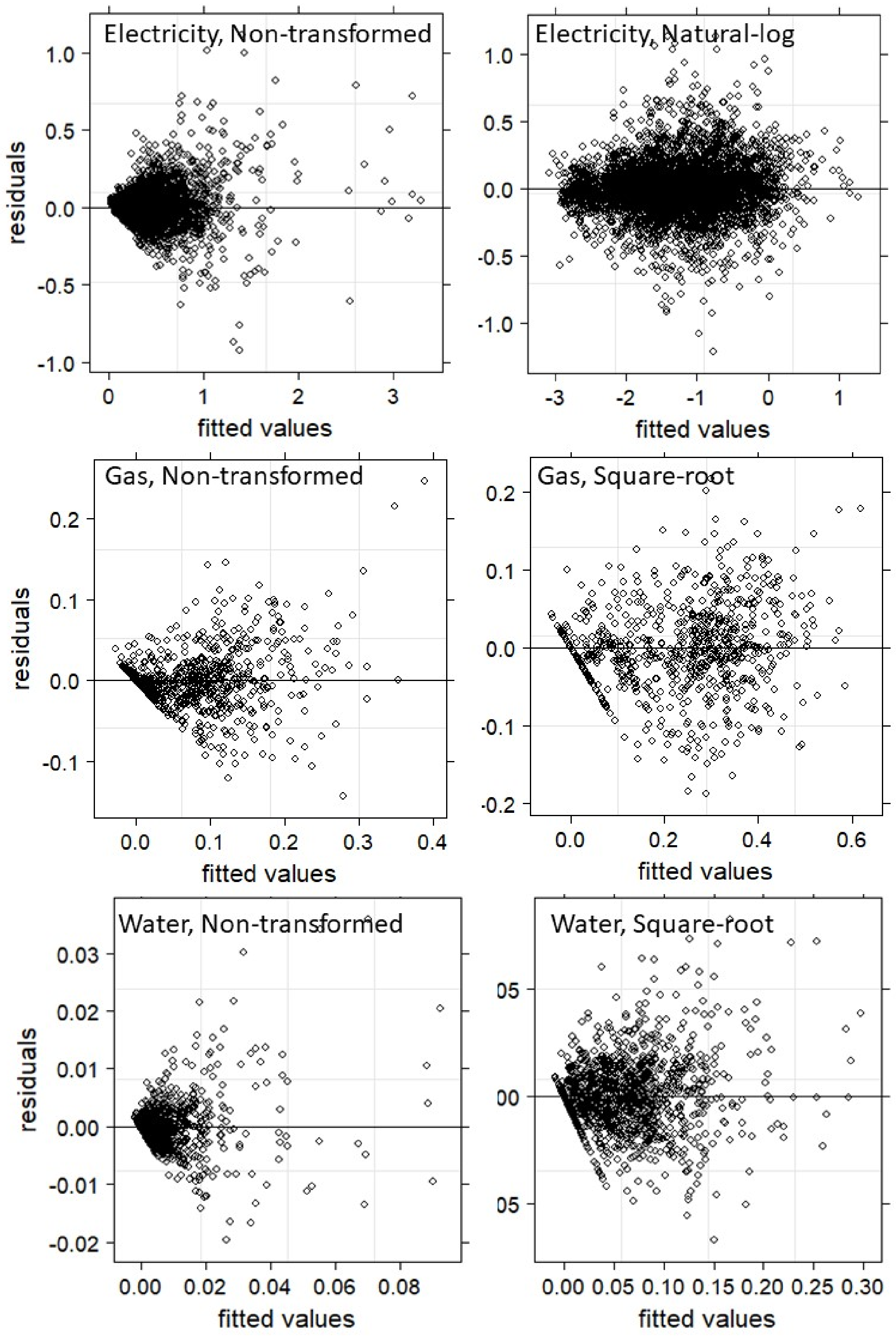

Figure S1: Residuals as a function of the values fitted by the interaction regression model containing both advice and instruction periods for the first lockdown, for non-transformed and transformed electricity, gas and water usage.
